# Population Characteristics and Clinical Outcomes from the Renal Transplant Outcome Prediction Validation Study (TOPVAS)

**DOI:** 10.3390/jcm11247421

**Published:** 2022-12-14

**Authors:** Sebastian Sallaberger, Lukas Buchwinkler, Susanne Eder, Stefan Schneeberger, Gert Mayer, Markus Pirklbauer

**Affiliations:** 1 Department of Internal Medicine IV—Nephrology and Hypertension, Medical University Innsbruck, 6020 Innsbruck, Austria; 2 Department of Visceral, Transplant and Thoracic Surgery, Medical University Innsbruck, 6020 Innsbruck, Austria

**Keywords:** kidney allograft, allograft rejection, Austrian kidney transplant cohort, non-living kidney transplant, immunosuppression, deceased donor kidney, deceased donor transplant, clinical transplantation outcomes

## Abstract

Kidney transplantation is the preferred method for selected patients with kidney failure. Despite major improvements over the last decades, a significant proportion of organs are still lost every year. Causes of graft loss and impaired graft function are incompletely understood and prognostic tools are lacking. Here, we describe baseline characteristics and outcomes of the non-interventional Transplant Outcome Prediction Validation Study (TOPVAS). A total of 241 patients receiving a non-living kidney transplant were recruited in three Austrian transplantation centres and treated according to local practices. Clinical information as well as blood and urine samples were obtained at baseline and consecutive follow-ups up to 24 months. Out of the overall 16 graft losses, 11 occurred in the first year. The patient survival rate was 96.7% (95% CI: 94.3–99.1%) in the first year and 94.3% (95% CI: 91.1–97.7%) in the second year. Estimated glomerular filtration rate (eGFR) improved from 37.1 ± 14.0 mL/min/1.73 m^2^ at hospital discharge to 45.0 ± 14.5 mL/min/1.73 m^2^ at 24 months. The TOPVAS study provides information on current kidney graft and patient survival, eGFR trajectories, and rejection rates, as well as infectious and surgical complication rates under different immunosuppressive drug regimens. More importantly, it provides an extensive and well-characterized biobank for the future discovery and validation of prognostic methods.

## 1. Introduction

The incidence and prevalence of kidney disease is increasing globally [1]. Kidney transplantation offers both a mortality, quality of life, and cost benefit in advanced kidney disease, irrespective of age, when performed in well selected individuals [2,3]. Several factors influence patient and graft survival, including recipient age, recurrence of primary disease, HLA matching, dialysis vintage, comorbidities, and most prominently donor organ quality [4,5,6]. Maintaining organ function with adequate immunosuppression leads to increased graft and patient survival due to decreased rejection rates [7,8]. Nevertheless, despite improved outcomes in recent years, a significant amount of kidney grafts (2.5–5%) is still lost annually [9,10]. Measures to improve graft survival are paramount to decrease the number of patients on or returning to dialysis as well as the time spent on a waiting list, and thus, to improve both the quality and length of transplant recipients’ life. In the first year after transplantation, kidney graft failure primarily results from acute rejection (17%), vascular (26%), or technical complications (12%), whereas thereafter, chronic allograft nephropathy is the main cause of graft failure (63%) [11]. Death with a functioning graft in the first year after transplantation is mainly caused by cardiovascular disease and infections (31% each). While malignancy accounts for only 7% of deaths with a functioning graft in this period, it is the leading cause thereafter (29%), followed by cardiovascular disease (23%) and infections (12%) [11].

Today, different immunosuppressive drug regimens have been established to prevent acute and chronic rejection and are adjusted to the expected immunological rejection risk. Patients with a low-risk for rejection have few to no HLA-mismatches and are non-sensitized prior to organ or tissue transplantations [8]. Glucocorticoids are the mainstay for induction (high-dose) and maintenance (low-dose) therapy in most kidney transplant recipients. Due to the well-known side effects of glucocorticoid-therapy, low-dose or steroid-free drug regimens were sought by both patients and physicians. Although well-controlled studies showed increased rejection rates in steroid-sparing or steroid-free groups, similar allograft function and survival rates, in addition to improved cardiovascular risk profiles, were documented in the steroid-sparing drug regimens [12,13].

Allograft loss and rejection can be further reduced using a T-cell depleting and modulating induction therapy [14,15,16]. While several induction therapy options are available, only two classes of drugs are frequently used based on patients’ immunological risk: anti-thymocyte globulin (ATG) for recipients with a high immunological risk [8,17] and anti-interleukin 2 receptor antibodies (IL2RA), which are recommended for patients with a low immunological risk [18,19,20,21]. In order to prevent rejection events and preserve graft function, appropriate maintenance immunosuppressive therapy is crucial. Today, the combination of two out of five agents with or without glucocorticoids is used: Calcineurin inhibitors (CNIs), mycophenolic acid (MPA)/mycophenolate mofetil (MMF), azathioprine, mTOR inhibitors, and Belatacept. Over the last decades, the combination of a CNI with MMF has emerged as the mainstay for maintenance immunosuppression with or without prednisone [7]: Cyclosporine was the first available CNI that revolutionized organ transplantation. Several years later, the use of another CNI, namely Tacrolimus, further improved graft survival and function as well as rejection rates. Moreover, the combination with MMF compared to Azathioprine also demonstrated to be beneficial regarding rejection rates [22,23,24,25,26,27]. Unfortunately, CNIs are, per se, nephrotoxic and long-term use contributes to allograft function decline and ultimately graft loss [28]. Therefore, different CNI-sparing regimes were investigated in the past, mainly focusing on minimal dosing, weaning or elimination after a certain time point, in combination or substitution with another immunosuppressant, or avoidance from transplantation onwards [8]. Here, we present baseline characteristics and clinical outcomes of 241 first or second kidney transplant recipients that were recruited in the Renal Transplant Outcome Prediction Validation (TOPVAS) study that aims at establishing and validating molecular models of kidney transplant outcome.

## 2. Materials and Methods

TOPVAS is a two-part non-interventional national multicenter study in patients undergoing a first or second deceased donor kidney transplant. In the observation part of the study, a prognostic panel of molecular biomarkers was derived from a set of transcriptomic profiles from zero-hour transplant kidney biopsies of 72 transplant recipients and correlated with clinical follow-up data to predict graft survival [29]. In the validation part, a cohort of 241 first or second kidney transplant recipients was recruited between Q4/2015 and Q1/2018 in three Austrian transplant centres (110 in Innsbruck, 15 in Linz and 116 in Vienna) and followed for two years. Immunosuppression was based on Tacrolimus and/or MMF with other agents administered according to local practices. Clinical and laboratory data were collected and recorded in a web-based database at the time of transplantation, at the day of hospital discharge, and at three, twelve, and 24 months after transplantation, respectively. Baseline zero-hour kidney tissue biopsies, as well as blood and urine specimens collected at baseline and at follow-up visits, were stored in a biobank to evaluate prognostic and predictive biomarkers and validate molecular models of kidney transplant outcome derived from the first part of the study. Studies on the predictive power of kidney biopsy evaluation and selected sets of biomarkers concerning kidney transplant outcome will be conducted in the near future. Here, we report baseline characteristics and clinical outcomes of the study population.

Patients over the age of 18, awaiting their first or second kidney transplant and with a panel reactive antibody frequency below 10% were informed about the study, and written informed consent was obtained. Patients were included in the study when none of the following exclusion criteria were met: Immunosuppressive regimen not containing either Tacrolimus or MMF at three months after transplantation, multi-organ, and living donor transplantation. Due to the non-interventional design, patients were eligible to participate in other prospective randomized trials.

The primary endpoint for the non-interventional prospective cohort study within TOPVAS is kidney transplant outcome (graft and patient survival) as well as validating the prognostic potential of candidate biomarkers, utilizing models of kidney transplant outcome derived from the first part of the study. Secondary outcomes are the incidence of clinically suspected and treated and/or biopsy proven acute and/or chronic rejection, delayed graft function, infections (cytomegalovirus and BK-Virus), post-transplant diabetes mellitus (PTDM), and surgical complications potentially affecting allograft function. A Major Adverse Cardiovascular Event (MACE) was defined as non-fatal myocardial infarction, heart failure, non-fatal stroke, peripheral amputation, or death due to cardiovascular disease. A non-functioning kidney graft was defined as dialysis dependence or creatinine clearance ≤ 20 mL/min at 3 months post-transplantation.

In order to report all data accurately, completely and transparently, the Strengthening the Reporting of Observational Studies in Epidemiology (STROBE) initiatives recommendations were followed [30]. The baseline population characteristics are reported as mean and standard deviation (sd) for normally distributed continuous data, skewed continuous data are reported as median and interquartile range (IQR). Categorical variables are reported as absolute and relative frequencies. Survival probabilities were calculated using the Kaplan–Meier method [31]. Analysis was conducted in R, version 4.1.1 (R Foundation for Statistical Computing, Vienna, Austria, 2022), and figures were produced using the survminer package (version 0.4.9, Kassambara, 2022) and the ggplot2 package (version 3.3.6, Pedersen, 2022).

The study was conducted in accordance with the World Medical Association Declaration of Helsinki. Written informed consent was obtained from each study participant prior to study, inclusion using a standardized patient information and consensus form according to Good Clinical Practice (GCP) guidelines, and the study protocol was approved by the ethics committees of the Innsbruck Medical University (Study ID AN2015-0101 348/4.25 357/5.15 (3745a)), Vienna Medical University (Study ID 1619/2015), and Upper Austria (Study ID E-38-15), prior to study initiation. All patient associated information was managed entirely coded. Data collection was conducted using an electronic evaluation form (case report form) according to GCP recommendations. All patient samples as well as clinical data are subject to privacy protection, according to the current European General Data Protection Regulation, the Health Insurance Portability and Accountability Act (HIPAA), and Research Ethics Board guidelines and recommendations.

## 3. Results

The baseline characteristics of patients at time of transplantation are shown in Table 1. The mean recipient age at time of transplantation was 55.88 ± 12.97 years, and 29.46% of recipients were female. All transplant recipients were exclusively Caucasian. Hypertension was prevalent in 223 (92.92%) patients, 102 (45.50%) had some form of cardiovascular disease, 50 (20.75%) patients had type 2 diabetes mellitus, and 22 (9.13%) patients had a history of malignancy. A total of 204 (84.65%) patients received their first kidney transplant. Among the 204 first kidney transplant recipients, 20 (9.8%) had panel reactive antibodies ≥1% (median 2%, IQR 2%). Moreover, 21 out of 37 (56.76%) second allograft recipients had panel reactive antibodies ≥1% (median 8%, IQR 13%). The average total dialysis vintage before kidney transplantation was 4.14 ± 3.82 years. Furthermore, no pre-emptive kidney transplantation was performed among the study population. Additionally, 210 (87.14%) patients received an IL2 receptor antagonist as induction therapy while ATG was utilized in 30 (12.44%) patients.

In this study, glomerulonephritis was the leading cause for renal replacement therapy (49 patients, 20.33%), followed by type 2 diabetic kidney disease (29 patients, 12.03%), hypertensive kidney disease (27 patients, 11.20%), and hereditary kidney disease (20 patients, 8.30%), details are provided in Table 2.

Donor characteristics are summarized in Table 3. Average donor age was 55.37 ± 16.60 years and 44.40% of donors were female. A total of 65 (26.97%) transplant recipients were older than 65 years. Expanded donor criteria were fulfilled in 153 (63.49%) donors: 93 (38.59%) donors were older than 60. A total of 60 (24.90%) donors were aged between 50 and 60 years and fulfilled a minimum of two of the following criteria: Positive history for hypertension, cerebrovascular death, and serum creatinine at transplantation ≥1.5 mg/dL. Hypertension was prevalent in 54 (22.41%) donors, and 16 (6.64%) donors had type 2 diabetes mellitus. Cerebrovascular disease was the leading cause of death (200 donors, 82.99%) and vasopressants were required in 167 (69.29%) donors, of which four were non-heart-beating donors. A total of 165 (68.46%) donors were seropositive for CMV, and 53 CMV seronegative recipients received an allograft from a CMV seropositive donor.

In total, no mismatches were present in 16 (6.64%) patients, one in 14 (5.81%) patients, two in 37 (15.35%) patients, three in 86 (35.68%) patients, four in 57 (23.65%) patients, five in 18 (7.47%) patients, and six in 12 (4.98%) patients. Details on the immunosuppressive regimen are shown in Table 4. Most patients received glucocorticoids throughout the study. The dose was tapered over the first few months to a maintenance dose of 5 mg per day. Likewise, the CNI doses were adapted over the course of the study according to international guidelines [18].

Details on relevant clinical outcomes are given in Table 5. Acute rejections and surgical complications occurred predominantly in the period shortly after transplantation. The predominant surgical complications were hematomas (*n* = 18), followed by lymphoceles (*n* = 10), as well as ureter necrosis and leakage (*n* = 5, each).

**Figure 1:**
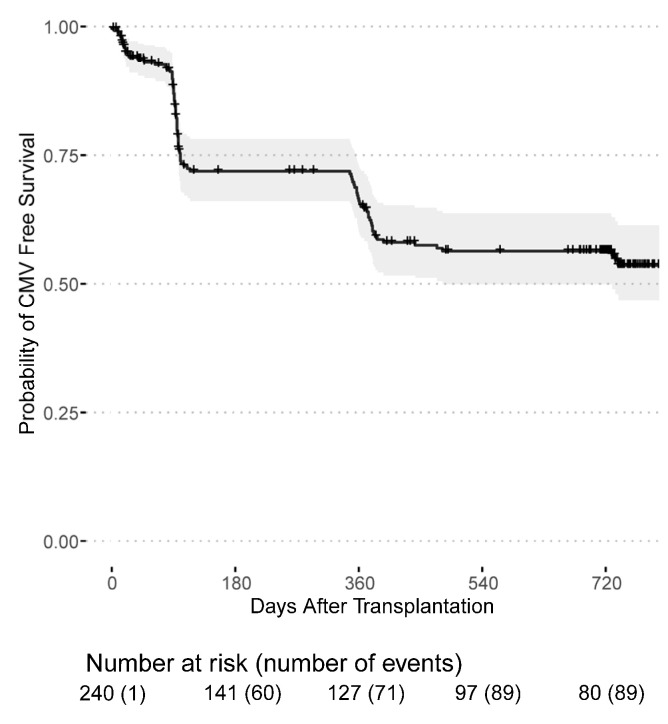
CMV infection-free survival probability for patients receiving a first or second kidney transplant from the day of transplantation.

**Figure 2:**
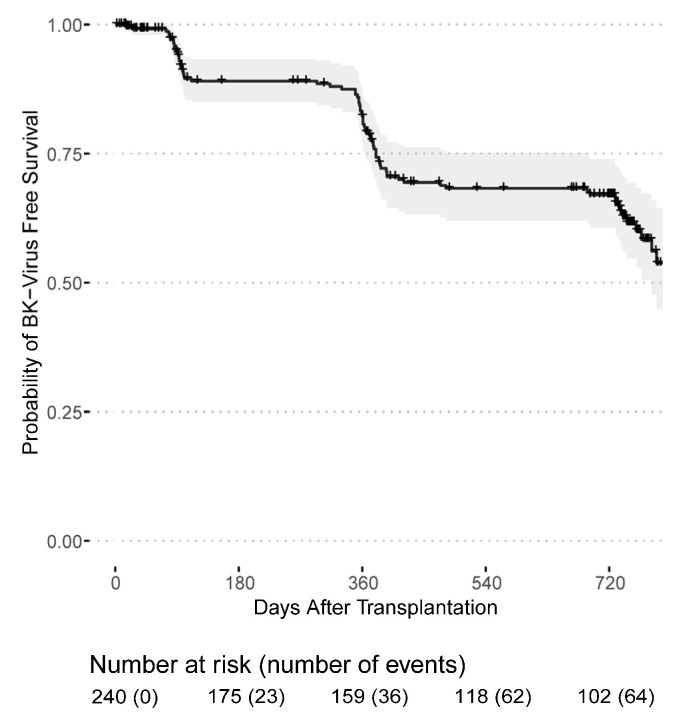
BK-Virus infection-free survival probability for patients receiving a first or second kidney transplant from the day of transplantation.

Infections with cytomegalovirus and BK-Viruses occurred mainly during the first year after transplantation. Among 94 CMV infections that were first documented after transplantation, 71 (75.5%) occurred in CMV seropositive recipients (164 patients), while 23 (24.5%) occurred in previously CMV seronegative recipients (77 patients). Thus, 46% of seropositive recipients and 29.87% of previously seronegative recipients developed a CMV infection during the follow-up period. Out of 23, 21 (91.3%) CMV seronegative recipients developing a CMV infection post transplantation received an allograft from a seropositive donor. The majority of post-transplant CMV infections were purely serology-confirmed, only three patients had clinically active CMV disease. Fortunately, no kidney grafts were lost due to CMV- or BK Virus-related infection. The death-censored CMV infection-free survival probability was 65.1% (95% CI: 58.8–71.9%) in the first year and 55.5% (95% CI: 49.0–63.0%) in the second year after transplantation (Figure 1). The death-censored BK-Virus infection-free survival probability was 79.1% (95% CI: 73.7–85.0%) in the first year and 65.5% (95% CI: 59.0–72.7%) in the second year (Figure 2).

PTDM was diagnosed mostly in the first year after transplantation. In total, 13 patients suffered a non-lethal MACE in the follow-up period: Five patients had a heart failure episode and five patients experienced myocardial infarction, three patients were not further specified.

There is a trend towards an increase in newly diagnosed malignant diseases over the course of the study.

**Figure 3:**
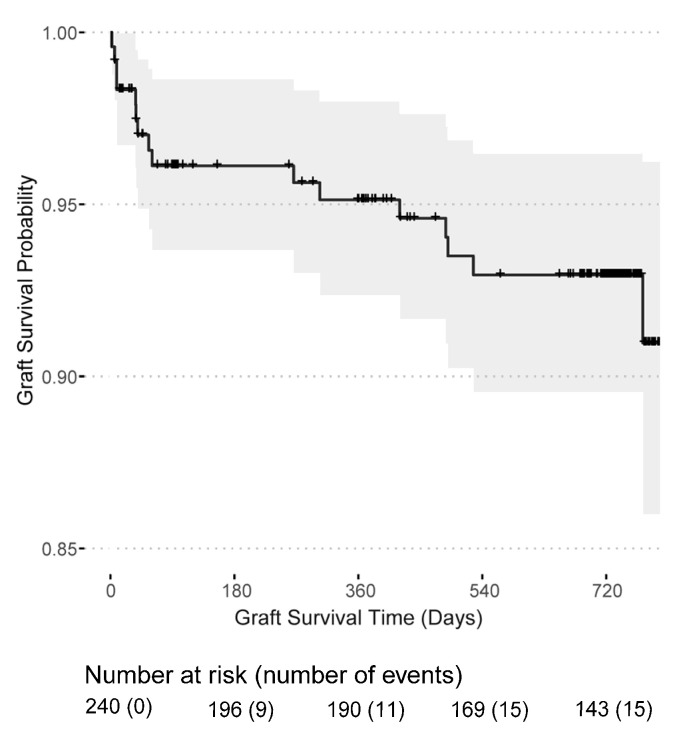
Kidney graft survival probability for patients receiving a first or second kidney transplant from the day of transplantation.

**Figure 4:**
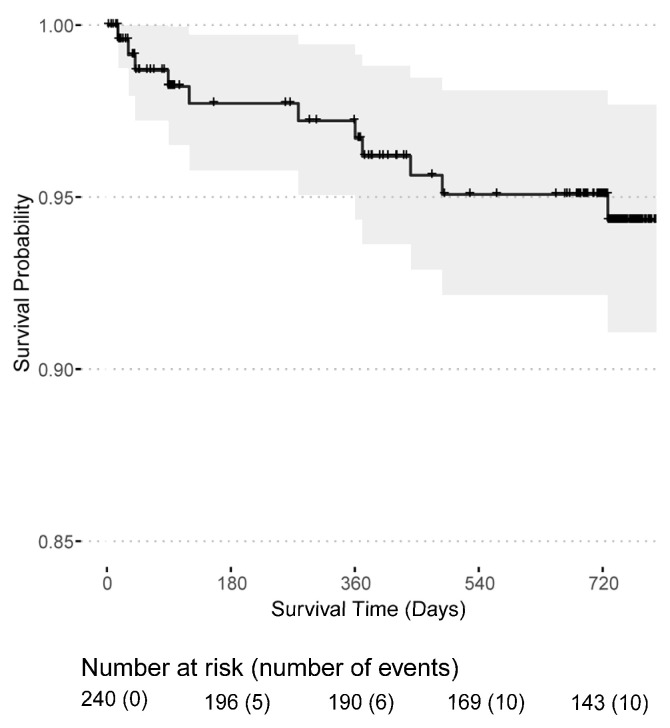
Patient survival probability for patients receiving a first or second kidney transplant from the day of transplantation.

Post-transplant dialysis was required in 90 (37.34%) patients and 9 (3.73%) patients remained with a non-functioning kidney graft, see Table 1. Surgical kidney graft removal was necessary in two transplant recipients, whereas the majority had an immunological graft loss. Graft losses occurred mainly in the first year (11 out of 16) after transplantation and two patients experienced a graft loss shortly before death. The death-censored graft survival probability was 95.1% (95% CI: 92.4–98.0%) in the first year and 92.9% (95% CI: 89.5–96.5%) in the second year after transplantation (Figure 3). Likewise, the mortality rate was highest in the first year after transplantation with a patient survival probability of 96.7% (95% CI: 94.3–99.1%) in the first year and 94.3% (95% CI: 91.1–97.7%) in the second year (Figure 4). In total, 16 kidney grafts were lost, and 12 transplant recipients died. Overall, 50 patients were lost to follow-up.

Selected laboratory and clinical data are depicted in Table 6. Initial anemia, most likely due to surgery related blood loss and chronic kidney disease, significantly ameliorated in the first three months after transplantation. Likewise, serum creatinine, serum urea, eGFR, and the urine protein/creatinine ratio continuously improved after transplantation. HbA1c levels greater than 6.5 mg/dL were present in 10 transplant recipients at hospital discharge, 42 recipients at three, 25 recipients at 12 and 29 recipients at 24 months follow-up, respectively.

## 4. Discussion

Patients receiving their first or second kidney transplant in one of three Austrian transplant centres (Innsbruck, Linz and Vienna) were recruited and followed for two years in the prospective observational, non-interventional, national, multicenter TOPVAS study. Here, we present the baseline characteristics and clinical outcomes of 241 kidney allograft recipients that were primarily treated with a Tacrolimus and MMF containing immunosuppressive regimen.

Data on patient and graft survival as well as other clinical outcomes (e.g., eGFR trajectories, rejection episodes as well as infectious and surgical complications) are provided for the first two years after transplantation. Based on the recruitment method and study design these data are highly representative for daily clinical practice. Today, kidney transplant recipients in industrialized countries are included in large clinical registries like the Austrian Dialysis and Transplantation registry or the United States Renal Data System (USRDS). However, clinical data included in these databases are often limited due to administrative demand. Therefore, the present study provides additional clinical information for patients in the first two years after kidney transplantation receiving an immunosuppressive drug regimen primarily based on Tacrolimus and/or MMF. Due to the non-interventional study design, immunosuppression was adjusted according to the clinical requirements and circumstances (e.g., dose reduction of MMF or change to a different drug class in case of profound leukopenia). Several studies have been investigating different immunosuppressive drug regimens and dosages to improve kidney graft and patient survival. Foremost, the Efficacy Limiting Toxicity Elimination (ELITE)-Symphony Trial offered important clinical insights into different immunosuppressive drug regimens including Tacrolimus/MMF after one and three years [24,32]. Immunosuppression with Tacrolimus and MMF offered the best overall kidney function and less biopsy-proven acute rejections [24,32]. In our study the rate of suspected and treated acute graft rejections was similar in the first year when compared with the (ELITE)-Symphony Trial population (17.4% vs. 17.2% respectively) [24,32].

Compared to unadjusted USRDS and European Renal Association – European Dialysis and Transplant Association (ERA-EDTA) Registry data, death-censored graft survival probability in the first year after deceased donor kidney transplantation is higher in our study population (92.5% vs. 91.3% vs. 95.1%, respectively) [9,32]. However, overall unadjusted patient survival in the TOPVAS study cohort is similar compared to both the US and European population, with 96.7% vs. 96.3% (96.1–96.5) vs. 96.7% (94.3–99.1), respectively [9,33]. While the unadjusted two-year graft survival rate is higher in the present study compared to the European deceased donor transplant recipient population, i.e., 92.9% (89.5–96.5) vs. 88.3% (88.0–88.6), respectively, the unadjusted two-year overall survival rates are similar between these two study populations, i.e., 94,3% (91.1–97.7) vs. 94.2% (94.0–94.5), respectively [33]. No statistically significant effect of dialysis vintage, HLA-mismatch and/or expanded donor criteria kidney grafts on graft survival was detected over the two-year follow-up (data not shown), which might be due to the limited observation period and/or population size. In our present study, the rate of acute rejection during the first year after transplantation was similar compared to previous studies [34,35,36]. Donor specific antibody formation, a risk factor for reduced kidney graft survival, was shown to be higher in patients with lower Tacrolimus trough levels in the first year after transplantation, and especially in patients with a higher immunological risk [37,38]. Similar results were also shown in a trial of extended-release Tacrolimus in low-immunological-risk kidney transplant recipients that were followed for one year [39]. In this study, donor specific antibodies with a mean fluorescence intensity (MFI) greater than 2000 were present in only one patient who had a Tacrolimus trough level below 7 µg/L at month three after transplantation. Donor specific antibodies were screened when rejection was clinically suspected. The Tacrolimus trough levels in this study were highest at the time of discharge, and the doses were subsequentially tapered during following visits to, achieve target trough levels according to the recommended thresholds reported in the second consensus report of the immunosuppressive Drugs Scientific Committee of the International Association of Therapeutic Drug Monitoring and Clinical Toxicity (IATDMCT) [40].

While 75.5% of post-transplant CMV infections occurred in previously CMV seropositive recipients, 24.5% of CMV infections were found in previously CMV seronegative recipients, predominantly resulting from seropositive donor allografts (21 of 23 cases). This is in line with data reported in the literature [41]. CMV prophylaxis and treatment was performed according to KDIGO recommendations [18].

PTDM is a serious complication as it may fuel kidney function decline by the development of diabetic kidney disease, and poses as an additional risk factor for metabolic disorders and infections [42]. Furthermore, it is associated with premature death and cardiovascular morbidity and mortality [43,44,45,46]. With the use of a high dose glucocorticoid drug regimen in the initial post-transplant period, PTDM develops in 4 to 27% of kidney transplant recipients, predominantly in the first three months after transplantation [42,47]. Besides glucocorticoids, Tacrolimus has the highest diabetogenic potential compared to other immunosuppressants [48,49,50,51]. In a Portuguese study, the cumulative incidence of PTDM was 21.3% and 24.8 at month 3 and 12, respectively. Incidence rates as well as primary occurrence in the first three months were similar when compared with our patient population [52].

Cardiovascular disease is one of the major causes of death in the kidney transplant recipient population [53,54]. The risk for cardiovascular events is higher compared to the general population and the incidence is highest in the first three months after transplantation [55]. In a Dutch retrospective cohort study, the incidence of a MACE in the first three months, the first, and the second year after transplantation was higher compared to our study with 4.1%, 7.6%, and 4% vs. 3%, 4%, and 2%, respectively [55]. However, in contrast to the current standards, in the Dutch study cohort extensive cardiovascular risk screening (e.g., stress echocardiography, scintigraphy or coronary angiography) was only performed in patients with diabetic kidney disease, positive cardiac history or abnormal routine evaluation (ECG, medical history, and physical exam) [55].

Compared to data regarding the primary disease leading to dialysis in the Austrian Dialysis and Transplantation registry, patients with glomerulonephritis are overrepresented and patients with vascular or diabetic kidney disease are underrepresented in this study [56]. In the US, the rate of glomerulonephritis leading to transplantation was similar to our study, yet diabetic and hypertensive kidney disease are more prevalent in the US, present in more than 50% of the patient population [9]. In this study the underlying kidney disease is not well categorized (“other disease”) in 37% of patients and therefore the true scale of different disease entities leading to dialysis and transplantation may differ significantly. However, due to the eligibility criteria and study recruitment protocol (all patients on the waiting list were informed of the study), the study population should reflect the general Austrian kidney transplant recipient population well.

The previously described disparity of men and women in the access to deceased donor kidney transplantation might also account for the present study population, as significantly more men received a kidney transplant [57,58].

The initial minimal recruitment count was met; however, more patients dropped out and were lost to follow-up than expected, being a limitation of the current study. The phenomenon of many patients choosing to perform follow-up visits at the outpatient nephrology clinic of their local hospital, instead of the transplant centre, may serve as a potential explanation. The data of these respective follow-up visits was not available for further work-up and inclusion in the study. To account for this attrition bias, we provided the number of all active patients at the time of visit.

This study was carried out before the SARS-CoV-2 pandemic. Current outcome data of transplant recipients infected with SARS-CoV-2 is conflicting: The majority of studies show a high mortality rate in transplant recipients hospitalized due to COVID-19, especially in the first months after transplantation, and in the elderly [59,60]. However, a recent study demonstrated that the outcome of COVID-19 in kidney transplant recipients is primarily dependent on concomitant co-morbidities and not on the immunosuppressive drug regimen, compared to matched controls [61]. As the SARS-CoV-2 pandemic is still ongoing and new variants are emerging, which differ in clinical severity and infectiousness, the impact on kidney transplant recipients needs to be addressed in further studies.

Besides therapeutic drug monitoring, different strategies to tailor the immunosuppressive therapy are currently under investigation. Several biomarker driven approaches for immunological and rejection monitoring already show promising results [10,62,63,64,65]. For example, the analysis of torque teno virus (TTV) load, a highly prevalent and non-pathogenic virus, emerged as a new tool to fine tune immunosuppression [66,67,68,69,70]. However, single biomarkers might be insufficient to represent the complex biophysical and immunological environment within the (kidney) graft. With the advent of large multi -omics registries, the transplant physician might get access to more accurate models of transplant outcome, which could aid post-transplant (immunosuppressive) management and improve patient care [71,72,73,74]. Models generated from specific transplant recipient populations usually offer a rather limited predictive validity in respect to other cohorts [75]. However, the predictive performance might be improved by using a rational subset of biomarkers across all cohorts [75]. Latest computational methods may offer new and promising perspectives for kidney medicine and transplantation [76,77]. For example, computer assisted image analysis of transplant kidney biopsies in another cohort identified markers predictive of graft loss [78,79]. Live confocal tissue assessment of kidney biopsies performed in this study population already offer insights on delayed graft function [80].

Therefore, combining sophisticated bioinformatic models with clinical (outcome) data and multiomics approaches, based on biobanked human samples, all of which are available for the TOPVAS study population, might stimulate further research and enable us to enter the age of precision transplantation medicine by individualizing (immunosuppressive) transplant recipient management.

## Figures and Tables

**Table 1:** Transplant recipient characteristics.

Characteristic	Value
Age (years *±* sd)	55.88 *±* 12.97
Sex transplant recipient (% female)	29.46
Height (cm *±* sd)	172.08 *±* 10.14
Weight (kg *±* sd)	80.02 *±* 17.47
BMI (kg/m^2^ *±* sd)	26.79 *±* 4.43
First kidney transplant (N, %)	204, 84.65
Positive CMV status (N, %)	164, 68.05
Need for dialysis post transplant (N, %)	90, 37.34
Primary non function (N, %)	8, 3.32
Induction therapy performed with:	
IL2 receptor antagonist (N, %)	210, 87.14
ATG (N, %)	30, 12.44
Plasmapheresis (N, %)	1, 0.41
Other (N, %)	2, 0.83
Medical history positive for:	
Hypertension (N, %)	223, 92.92
Cardiovascular disease (N, %)	102, 45.50
Diabetes mellitus (N, %)	50, 20.75
Malignancy (N, %)	22, 9.13

BMI: Body-mass-index, N: Number, sd: Standard deviation, CMV: Cytomegalovirus, IL2: Interleukine-2, and ATG: Anti-thymocyte globulin.

**Table 2:** Kidney disease leading to transplantation.

Characteristic	Value (N, %)
Glomerulonephritis	49, 20.33
Hypertensive kidney disease	27, 11.20
Hereditary kidney disease	20, 8.30
Type 2 Diabetic kidney disease	29, 12.03
Type 1 Diabetic kidney disease	6, 2.49
Systemic autoimmune disease	5, 2.07
Other disease	89, 36.93
Unknown	16, 6.64

N: Number.

**Table 3:** Donor characteristics.

Characteristic	Value
Donor age at transplantation (years ± sd)	55.37 ± 16.60
Sex donor (% female)	44.40
Dependent on vasopressants (N, %)	167, 69.29
Non-heart-beating (N, %)	20, 8.30
Serum creatinine at transplantation (mg/dL ± sd)	1.06 ± 0.76
Cold ischemia time (hours ± sd)	14.10 ± 5.48
Warm ischemia time (minutes ± sd)	30.09 ± 8.32
Positive CMV status (N, %)	165, 68.46
Medical history in donor positive for:	
Hypertension (N, %)	54, 22.41
Diabetes mellitus (N, %)	16, 6.64
Cause of donor death:	
Cerebrovascular (N, %)	200, 82.99
Other (N, %)	40, 16.60
Unknown (N, %)	1, 0.41

sd: Standard deviation, N: Number, CMV: Cytomegalovirus.

**Table 4:** Immunosuppression after transplantation.

Characteristic	Value			
	Follow-Up			
	Discharge	3 Months	12 Months	24 Months
Patients on corticosteroids (N, %)	227, 99.56	207, 97.64	173, 90.58	143, 86.67
Prednisone dose (mg, median, IQR)	20, 5	10, 3.5	5, 0	5, 0
Patients on Tacrolimus & MMF (N, %)	182, 79.82	173, 81.60	160, 83.77	126, 76.36
Patients on Tacrolimus (N, %)	205, 89.91	187, 88.21	173, 90.58	143, 86.67
Tacrolimus through level (µg/L ± sd)	9.36 ± 3.21	8.95 ± 3.41	7.32 ± 2.48	6.54 ± 2.15
Patients on MMF (N, %)	219, 96.05	197, 92.92	174, 91.10	145, 87.88
MMF dose (mg, median, IQR)	2000, 375	2000, 940	1440, 1000	1000, 1000
Patients on Cyclosporine A (N, %)	16, 7.02	15, 7.08	16, 8.38	14, 8.48
Patients on Azathioprine (N, %)	5, 2.19	8, 3.77	8, 4.19	8, 4.84
Patients on Belatacept (N, %)	7, 3.07	8, 3.77	10, 5.24	10, 6.06
Active patients at time of (N)	228	212	191	165

This table shows the immunosuppressive regimens used throughout the study. The number of active patients (alive with a functioning kidney transplant and not lost to follow-up) is shown in the last row. The proportion of patients on the respective drug is calculated using the number of patients taking the medication compared to all active patients. N: Number, MMF: Mycophenolate mofetil, IQR: Interquartile range, and sd: Standard deviation.

**Table 5:** Clinical Endpoints.

Characteristic	Value			
	Follow-Up			
	Discharge	3 Months	12 Months	24 Months
Treatment for rejection (N, %)	31, 12.86	34, 14.11	42, 17.43	44, 18.26
Biopsy proven rejection ^1^ (N, %)	12, 4.98	13, 5.39	17, 7.05	19, 7.88
Surgical complications (N, %)	30, 12.45	48, 19.92	62, 25.73	64, 26.56
CMV infection ^2^ (N, %)	16, 6.64	47, 19.50	57, 23.65	14, 5.81
BK-Virus infection ^2^ (N, %)	2, 0.83	22, 9.13	54, 22.41	35, 14.52
PTDM (N, %)	13, 5.39	33, 13.69	51, 21.16	60, 24.90
MACE (N, %)	4, 1.66	7, 2.9	9, 3.73	13, 5.39
Malignancy (N, %)	0, 0	2, 0.83	8, 3.32	15, 6.22
Graft loss (N, %)	9, 3.73	9, 3.73	11, 4.56	16, 6.64
Death (N, %)	4, 1.66	5, 2.07	8, 3.32	12, 4.98
Loss to follow-up (N, %)	1, 0.41	16, 6.64	32, 13.28	50, 20.75
Active patients at time of (N, %)	228, 94.6	212, 87.7	191, 79.3	165, 68.5

Clinical endpoints are added up during follow-up. Proportions are calculated using the total number of patients. The number of active patients (alive with a functioning kidney transplant and not lost to follow-up) at the time of the visit are shown in the last row. The number of patients lost to follow-up was added up during follow-up and the proportion calculated with all 241 patients included in the study. N: Number, CMV: Cytomegalovirus, PTDM: Post-transplant diabetes mellitus. MACE: Major adverse cardiovascular event. ^1^: Kidney biopsies with a BANFF Score greater than 1 were classified as a rejection episode. ^2^: Cytomegalovirus infections, confirmed by serology/PCR or active disease and BK-Virus infections, confirmed by PCR in serum and/or urine, in the respective follow-up visits are depicted.

**Table 6:** Selected laboratory and clinical measurements.

Characteristic	Value (Mean *±* SD)			
	Follow-Up			
	Discharge	3 Months	12 Months	24 Months
Systolic blood pressure (mmHg)	NA	135.75 *±* 14.50	134.91 *±* 14.55	133.86 *±* 13.42
Diastolic blood pressure (mmHg)	NA	79.96 *±* 9.70	79.00 *±* 9.42	78.37 *±* 8.90
Hemoglobin (g/dL)	9.66 *±* 1.37	12.01 *±* 1.83	13.28 *±* 2.05	13.57 *±* 1.90
Serum creatinine (mg/dL)	2.08 *±* 1.18	1.69 *±* 0.64	1.61 *±* 0.69	1.57 *±* 0.56
Serum urea (mg/dL) eGFR (mL/min/1.73 m^2^)	53.02 *±* 34.94 37.14 *±* 14.07	42.52 *±* 25.33 42.02 *±* 13.86	43.39 *±* 25.14 44.05 *±* 14.04	43.08 *±* 27.22 45.01 *±* 14.53
Fasting glucose (mg/dL)	105.00 *±* 32.24	122.60 *±* 58.89	115.76 *±* 41.11	118.99 *±* 50.81
HbA1c (%)	5.89 *±* 1.01	6.12 *±* 1.21	5.91 *±* 0.98	6.04 *±* 1.26
Urine proteine/creatinine ratio (mg/g, median, IQR)	285.5, 298.0	181.0, 224.25	138.5, 175.25	122.0, 176.0
Urine albumine/creatinine ratio (mg/g, median, IQR)	106.25, 169.75	48.00, 110.50	35.50, 80.50	29.00, 126.10

Selected laboratory and clinical measurements of all active transplant recipients at the follow-up visits are shown. Glomerular filtration rate was estimated (eGFR) using an isotope dilution mass spectrometry (IDMS) traceable creatinine measurement calculated with the MDRD-equation (Modification of Diet in Renal Disease). NA: No Data available, HbA1c: Hemoglobin A1c, IQR: Interquartile range.

## Data Availability

The data presented in this study are available on request from the corresponding author. The data are not publicly available due to data protection.
